# The inhibition of Bax activation-induced apoptosis by RasGRP2 via R-Ras-PI3K-Akt signaling pathway in the endothelial cells

**DOI:** 10.1038/s41598-019-53419-4

**Published:** 2019-11-13

**Authors:** Jun-ichi Takino, Takuma Sato, Kentaro Nagamine, Takamitsu Hori

**Affiliations:** 1 Laboratory of Biochemistry, Hiroshima International University, Hiroshima, Japan; 2 Department of Clinical Nutrition, Hiroshima International University, Hiroshima, Japan

**Keywords:** Apoptosis, Molecular biology

## Abstract

Apoptosis of endothelial cells is a very important event in various diseases and angiogenesis. We recently reported that ras guanyl nucleotide releasing protein 2 (RasGRP2), which is a guanine nucleotide exchange factor, was expressed in the human umbilical vein endothelial cells (HUVECs) and that Rap1 activation by its overexpression inhibited apoptosis by suppressing tumor necrosis factor-α induced-reactive oxygen species (ROS) production. However, other signaling pathways and roles of RasGRP2 not mediated via Rap1 are not well understood. Therefore, we compared the Mock (M) and the RasGRP2-stable overexpression (R) immortalized HUVECs using BAM7 and anisomycin, which are apoptosis inducers. BAM7 and anisomycin induced apoptosis without causing ROS production, and such apoptosis was significantly increased in M cells, but not in R cells. RasGRP2 suppressed BAM7- and anisomycin-induced apoptosis, but not via the Rap1 pathway as observed using Rap1 knockdown. Furthermore, RasGRP2 activated not only Rap1 but also R-Ras, and suppressed apoptosis by activating R-Ras-phosphoinositide 3-kinase (PI3K)-Akt signaling pathway. The phosphorylation of Akt by RasGRP2 inhibited Bax translocation by promoting translocation of hexokinase-2 (HK-2) from cytoplasm to mitochondria. Taken together, it was suggested that RasGRP2 suppresses the Bax activation-induced apoptosis by promoting HK-2 translocation to mitochondria via R-Ras-PI3K-Akt signaling pathway.

## Introduction

Vascular endothelial cells occur in the monolayer on the inside of blood vessels and act as a barrier to regulate the migration of blood components to the vessel wall. Vascular endothelial cells are influenced by various factors (for example, endoplasmic reticulum stress, hyperglycemia, methylglyoxal, oxidized low density lipoprotein, ischemia-reperfusion, tumor necrosis factor-α (TNF-α), and lipopolysaccharide), and often cause apoptosis^1–7^. Apoptosis of endothelial cells induced by endoplasmic reticulum stress and oxidized low density lipoprotein or by hyperglycemia and methylglyoxal, associated with diabetes, is involved in the development and progression of atherosclerosis^1,4–6^. In addition, apoptosis of endothelial cells by ischemia-reperfusion and inflammation results in further tissue damage^2,3^. On the other hand, it has also been reported to be associated with angiogenesis remodeling into a mature network^8^. Therefore, apoptosis of endothelial cells is a very important event in various diseases and angiogenesis.

Ras guanyl nucleotide releasing protein (RasGRP), which is a guanine nucleotide exchange factor (GEF), consists of four members, RasGRP1–4, and activates small GTPases, such as Ras and Rap. RasGRP family is composed of the Ras exchange motif, CDC25 domain, EF-hands, and C1 domain^9,10^. Among the RasGRP family, RasGRP2, also called CalDAG-GEFI, cannot bind to diacylglycerol via mutated C1 domain^11^ and is translocated by binding to F-actin via Ras exchange motif region^12^. GEF activity of RasGRP2 is controlled by calcium-dependent rearrangement of EF hands^13^. The main target of RasGRP2 is well known to be Rap1^9^. Several studies on the function of RasGRP2 and its mutation or deficiency have been reported in the platelets and leukocytes^14–19^. RasGRP2 is associated with immune-mediated thrombosis and thrombocytopenia^16^, with integrin-independent neutrophil chemotaxis^17^, and with regulation of platelet and T-cell adhesion via integrin^18,19^. On the other hand, RasGRP2 has been reported to be associated with rheumatic synovium of destructive arthritis^20^ and Huntington’s disease striatum^21^. In addition, most of these activities of RasGRP2 are mediated via activation of Rap1.

We also identified *xrasgrp2* (homolog of the human *rasgrp2*) as a novel vascular related gene in Xenopus embryos and showed that the vascular formation was induced by its overexpression, and conversely, the vascular development was delayed by its knockdown^22–25^. Furthermore, we showed that RasGRP2 is expressed in the human vascular endothelial cells, including the human umbilical vein endothelial cells (HUVECs)^26^, and that Rap1, activated by RasGRP2, suppresses apoptosis by suppressing reactive oxygen species (ROS) production via NADPH oxidase (NOX) inhibition in the endothelial cells^27^. These results suggested that RasGRP2 in the endothelial cells acts as a defense factor against apoptosis to maintain blood vessels. However, other signaling pathways and roles of RasGRP2 not mediated via Rap1 are not well understood.

Therefore, we performed further analysis of apoptosis-suppressing mechanism of RasGRP2 and discovered the R-Ras signaling pathway of RasGRP2 and suppression of apoptosis via inhibition of Bax translocation via its signaling pathway in the human endothelial cells.

## Results

### RasGRP2 inhibits apoptosis without the Rap1 signaling pathway

**Figure 1:**
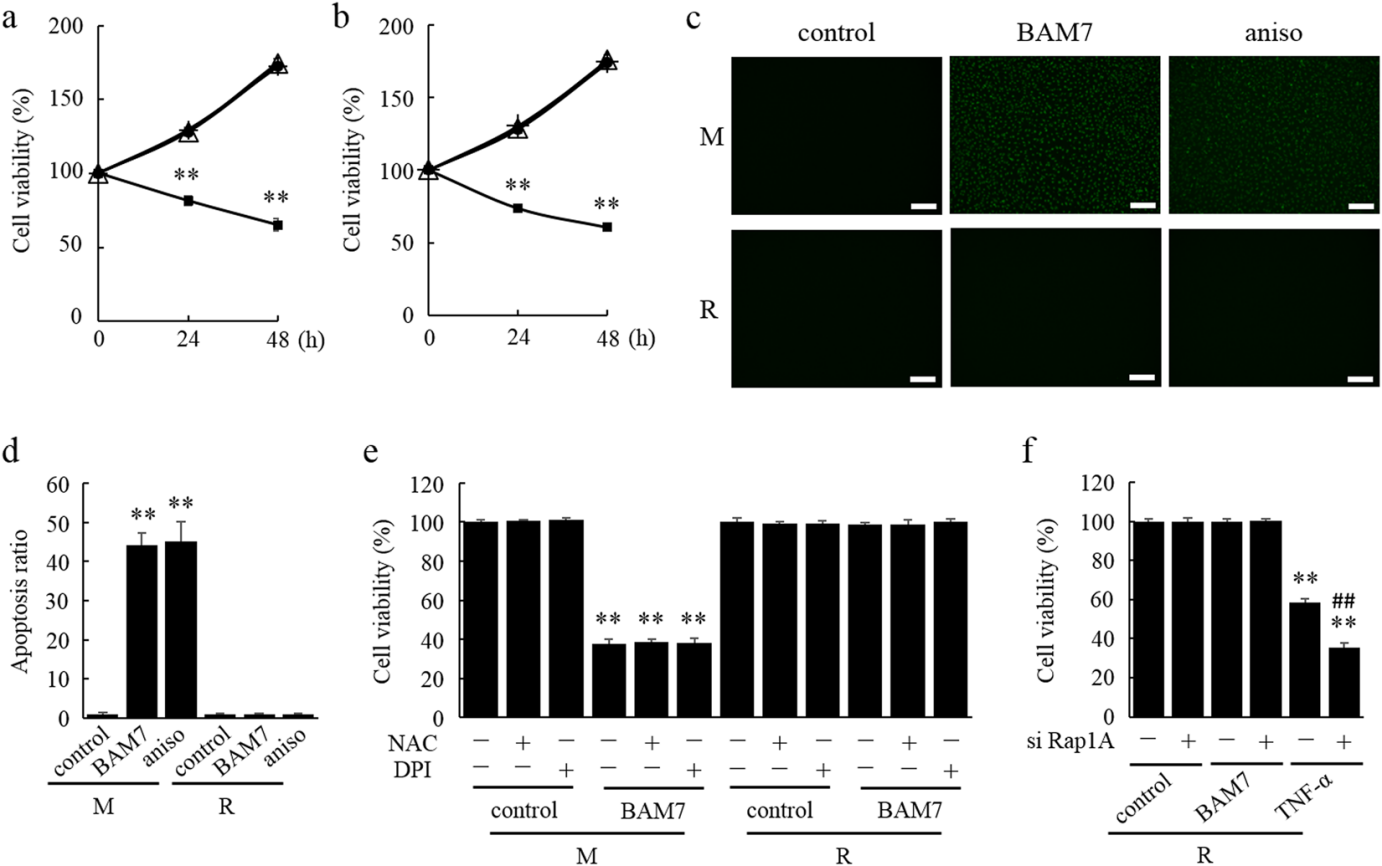
Induction of apoptosis without ROS production and its suppression by RasGRP2 without the Rap1 pathway. (**a**,**b**,**e**,**f**) Cell viability was determined by Cell Counting Kit-8 assay. Cells were incubated with or without BAM7 (**a**) or anisomycin (**b**) for 24 h to 48 h. Cell viability at 0 h was taken as 100%, circle: M control, square: BAM7- or anisomycin-treated M cells, triangle: R control, cross: BAM7- or anisomycin-treated R cells. (**c**) Apoptosis for 24 h was determined by NucView488; scale bar = 250 μm. (**d**) Apoptosis ratio in M cells control was taken as 1. (**e**) Cells were pre-incubated with or without NAC or DPI and incubated with or without BAM7 for 48 h. (**f**) Cells were pre-treated with siRNA against Rap1A or negative control siRNA and incubated with or without BAM7 or TNF-α for 48 h. M: mock cells, R: RasGRP2-stable overexpression cells, aniso: anisomycin. Data are shown as the mean ± SD (n = 3), **P < 0.01 compared with each cell control at the same time point, and ^##^*P < *0.01 compared with each R cell transfected with the negative control siRNA.

To investigate the effect of RasGRP2 on apoptosis in endothelial cells, we compared the cell viability of Mock (M) cells and RasGRP2-stable overexpression (R) cells using BAM7 and anisomycin, which are apoptosis inducers. The cell viabilities with or without BAM7 treatment for 24 h and 48 h were 127.8% ± 1.9% vs. 81.5% ± 3.2% (at 24 h, P < 0.01), and 172.6% ± 2.0% vs. 65.2% ± 4.1% (at 48 h, P < 0.01) in M cells, and 128.3% ± 2.7% vs. 129.5% ± 1.4% (at 24 h), and 174.6% ± 3.3% vs. 172.3% ± 2.1% (at 48 h) in R cells (Fig. 1a), respectively. With or without anisomycin, cell viabilities were 128.3% ± 2.7% vs. 73.6% ± 2.7% (at 24 h, P < 0.01), and 174.0% ± 3.1% vs. 60.5% ± 2.6% (at 48 h, P < 0.01) in M cells, and 128.9% ± 3.4% vs. 130.5% ± 1.4% (at 24 h), and 176.0% ± 3.7% vs. 174.0% ± 2.0% (at 48 h) in R cells (Fig. 1b), respectively. Thus, the cell viabilities were significantly decreased following treatment with BAM7 or anisomycin in M cells, but not in R cells. These results were similar for other clones (Fig. S1a,b).

Furthermore, apoptosis analysis was performed using a NucView 488 which detects the activation of caspase-3 to confirm whether the decrease in cell viability was due to apoptosis. Apoptosis was significantly increased after treatment with BAM7 or anisomycin in M cells, but not in R cells (Fig. 1c,d). The activities of caspase-3/7 were also significantly increased after treatment with BAM7 in M cells, but not in R cells (Fig. S2a). Similarly, cleaved poly (ADP-ribose) polymerase (PARP) was detected after treatment with BAM7 in M cells (Fig. S2b). Thus, BAM7 induced these proteins. The decrease in cell viability was inhibited by pre-treatment with Quinoline-Val-Asp-Difluorophenoxymethylketone **(**Q-VD-OPH), which is a pan-caspase inhibitor (Fig. S2a–c). Therefore, the decrease in cell viability was shown to be related to cell apoptosis. In addition, the decrease in cell viability by increase in apoptosis was not restored after pre-treatment with N-acetyl cysteine (NAC), which is a ROS scavenger, or diphenyleneiodonium (DPI), which is a NOX inhibitor (Fig. 1e, S3a). While the intracellular ROS was markedly increased after treatment with TNF-α as a positive control in M cells, it was not detected after treatment with BAM7 or anisomycin (Fig. S3b). These results, in addition to our previous report on suppression of apoptosis caused by ROS production via NOX activation^27^, showed that apoptosis mediated by BAM7 and anisomycin is not induced by ROS production in these cells and that RasGRP2 also suppresses the apoptosis pathway not mediated by ROS production.

Next, in order to confirm whether the RasGRP2-Rap1 pathway is involved in the suppression of apoptosis, we treated the cell with a small interfering RNA (siRNA) against Rap1. As a result, the siRNA against Rap1 markedly reduced the expression of Rap1 protein, and thus, the Rap1 activity of R cells was reduced to a level comparable to that of negative control siRNA-treated M cells (Fig. S3c). The decrease of cell viability following treatment with TNF-α as a positive control further reduced after knockdown of Rap1 in R cells. Whereas, despite the remarkable decrease of Rap1 activity after knockdown of Rap1 protein, the induction of apoptosis by treatment with BAM7 was completely suppressed (Fig. 1f). This indicated that RasGRP2 suppresses apoptosis but not via Rap1 signaling pathway.

### RasGRP2 activates R-Ras-phosphoinositide 3-kinase-Akt signaling pathway and inhibits apoptosis

**Figure 2:**
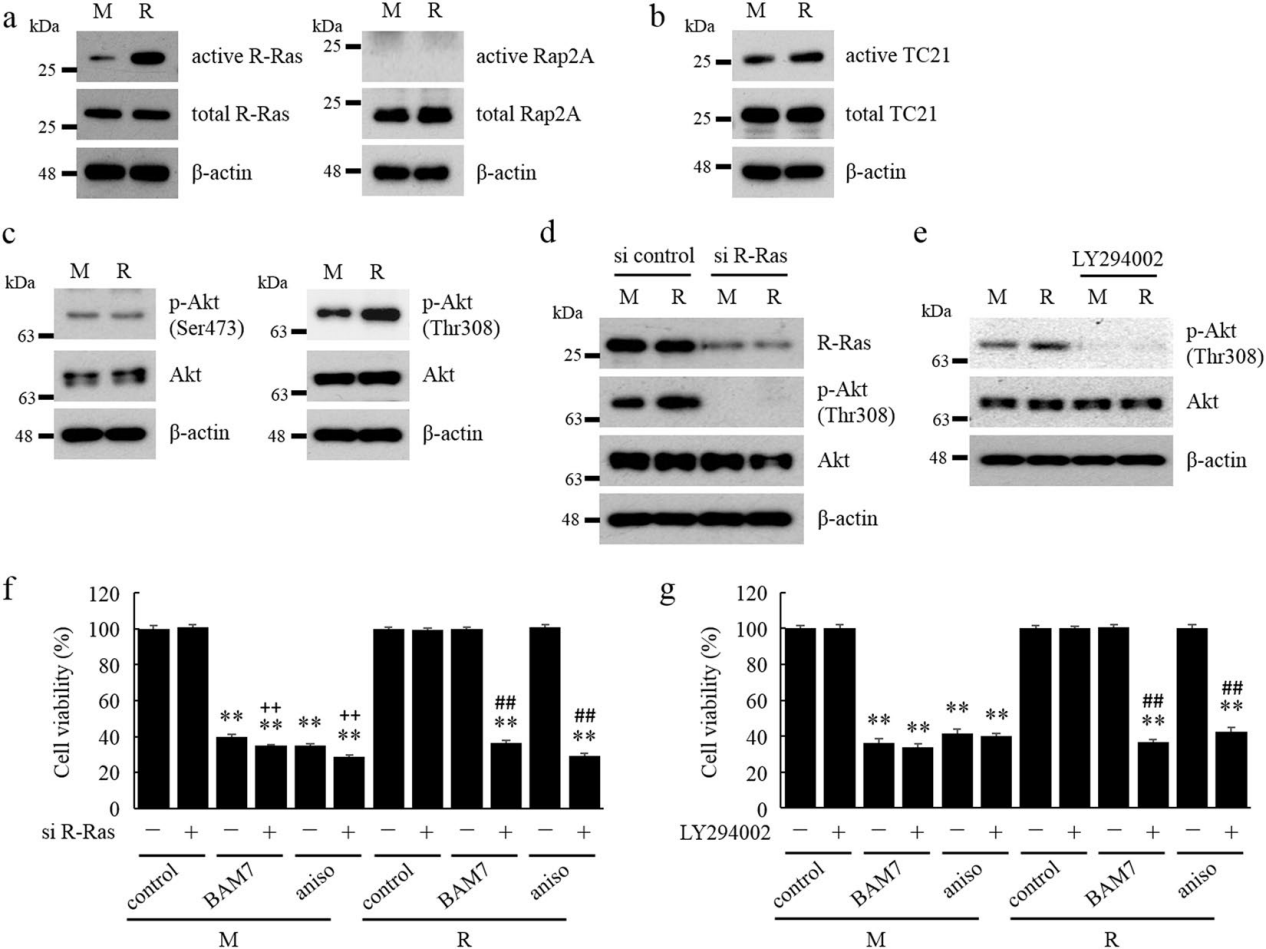
Activation of R-Ras-PI3K-Akt signaling pathway by RasGRP2 and suppression of apoptosis via its signaling pathway. (**a**–**e**) Each effect was detected by western blotting. (**a**) Activated R-Ras and Rap2A were isolated by pull-down assay using RalGDS-RBD agarose beads. (**b**) Activated TC21 was isolated by pull-down assay using Raf1-RBD agarose beads. (**c**–**e**) Phosphorylation of Akt was detected using specific antibodies. Cells were treated with siRNAs against R-Ras or negative control siRNA (**d**), or incubated with or without LY294002 (**e**). (**f**,**g**) Cell viability was determined by Cell Counting Kit-8 assay. (**f**) Cells were pre-treatment with siRNAs against R-Ras or negative control siRNA and incubated with or without BAM7 or anisomycin for 48 h. (**g**) Cells were pre-incubated with or without LY294002 and incubated with or without BAM7 or anisomycin for 48 h. M: mock cells, R: RasGRP2-stable overexpression cells, aniso: anisomycin. Data are shown as the mean ± SD (n = 3), **P < 0.01 compared with each cell control, ^##^*P < *0.01 compared with each R cell without pre-treatment or transfected with the negative control siRNA, and ^++^*P < *0.01 compared with each M cell transfected with the negative control siRNA.

We examined small GTPases other than Rap1 that are activated by RasGRP2 directly, using RalGDS-RBD and Raf1-RBD agarose beads. As a result, R-Ras, Ras-like protein TC21 (TC21), and Rap2A proteins were expressed in the endothelial cells, and Rap2A activity was not observed, and in R cells, TC21 activity was slightly increased and R-Ras activity was significantly increased (Fig. 2a,b). The expression level of interacting proteins did not change in response to RasGRP2 overexpression. Therefore, RasGRP2 was shown to mainly activate Rap1 and R-Ras in the endothelial cells.

Next, we examined the R-Ras signaling pathway. Although the phosphorylation status of Akt on Ser473 did not change significantly, that on Thr308 was increased in R cells compared to M cells (Fig. 2c). Furthermore, we examined whether the phosphorylation of Akt on Thr308 was dependent on R-Ras and phosphoinositide 3-kinase (PI3K) signaling using siRNAs against R-Ras and LY294002, which is a PI3K inhibitor. The phosphorylation of Akt on Thr308 was remarkably decreased by R-Ras knockdown in R cells (Fig. 2d), and a similar result was also obtained after LY294002 treatment (Fig. 2e). These results showed that RasGRP2-dependent increase in phosphorylation of Akt on Thr308.

In order to confirm whether the R-Ras-PI3K-Akt signaling pathway, activated by RasGRP2, is involved in the suppression of apoptosis, we examined the cell viability using siRNAs against R-Ras and LY294002. Figure 2f showed that the cell viability was significantly decreased after treatment with BAM7 or anisomycin along with R-Ras knockdown compared to by treatment with BAM7 or anisomycin in R cells. In addition, the reduction in cell viability was comparable to that of the treatment with BAM7 or anisomycin in M cells. A similar result was also obtained after LY294002 treatment (Fig. 2g). These results indicated that RasGRP2 suppresses apoptosis by activating R-Ras-PI3K-Akt signaling pathway. TC21 expression was analyzed in the same way; however, no effect was noted. (Fig. S4a,b)

### Endogenous RasGRP2 also activates R-Ras and inhibits apoptosis

**Figure 3:**
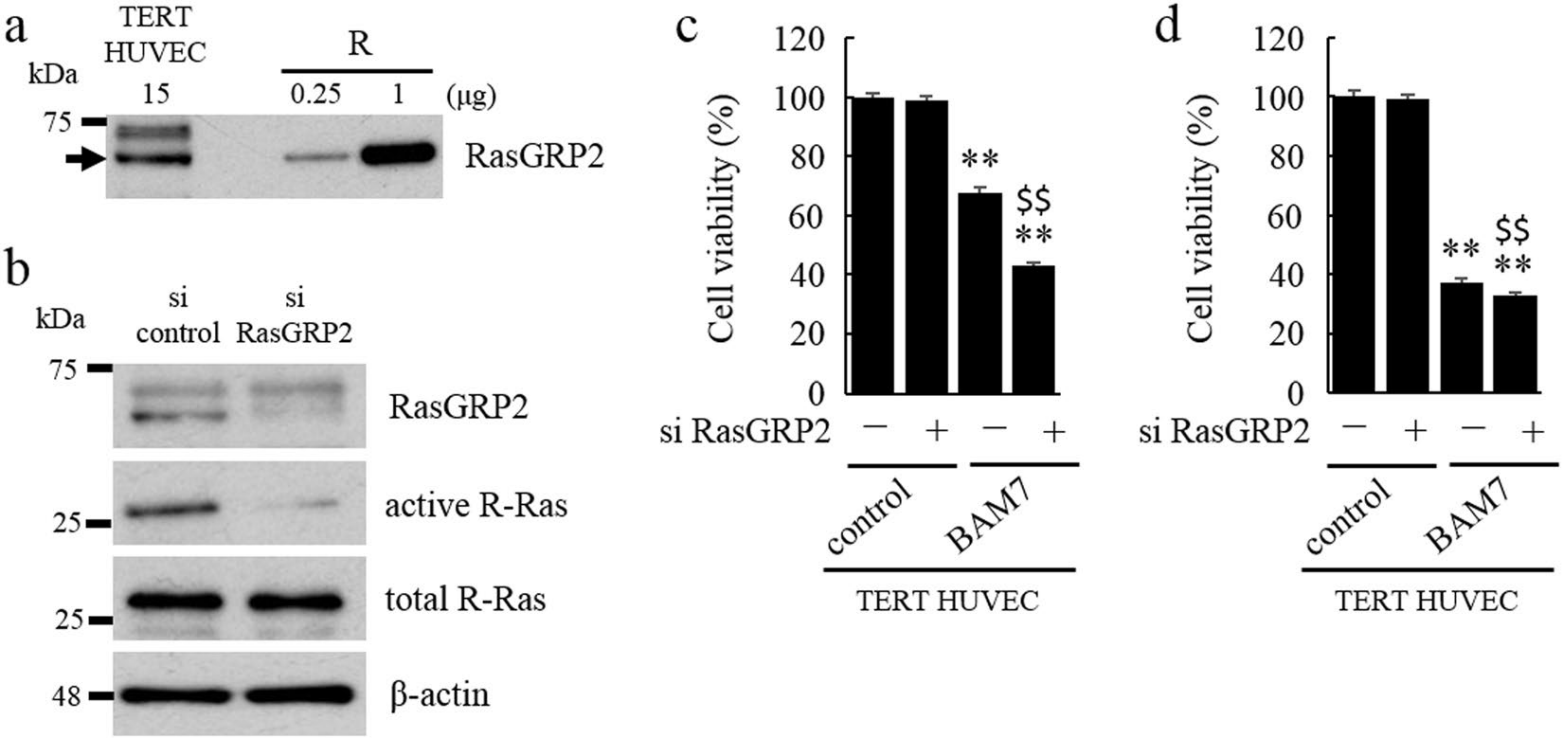
Expression of endogenous RasGRP2 in TERT HUVECs and suppression by endogenous RasGRP2 of the decreased cell viability. (**a,b**) Each effect was detected using western blotting. (**a**) RasGRP2 was detected using a specific antibody. R: RasGRP2-stable overexpression cells. (**b**) Activated R-Ras was isolated using a pull-down assay with RalGDS-RBD agarose beads. Cells were treated with siRNA against RasGRP2 or a negative control siRNA. (**c,d**) Cell viability was determined using a Cell Counting Kit-8 assay. Cells were pre-treated with an siRNA against RasGRP2 or a negative control siRNA and incubated with or without BAM7 (**c**: 10 μM, **d**: 20 μM) for 48 h. Data are shown as the mean ± SD (n = 3), **P < 0.01 compared with each cell control, and ^$$^*P < *0.01 compared with each cell transfected with the negative control siRNA.

To determine whether endogenous RasGRP2 shows similar behavior to exogenous RasGRP2, we detected the expression of endogenous RasGRP2 in the TERT HUVECs, and examined R-Ras activity and cell viability using siRNAs against RasGRP2. Endogenous RasGRP2 is expressed in the TERT HUVECs (Fig. 3a), and the R-Ras activity was reduced by knockdown of endogenous RasGRP2 expression (Fig. 3b). In TERT HUVECs with RasGRP2 knockdown treated with BAM7, cell viability was significantly decreased compared with that in BAM7-treated, control siRNA-transfected cells (Fig. 3c). Thus, knockdown of endogenous RasGRP2 increased BAM7-induced apoptosis. Furthermore, RasGRP2 knockdown also slightly decreased the viability of cells treated with BAM7 (20 μM), a condition similar to transfected cells, compared with that in control siRNA knockdown (Fig. 3d). The degree of this decrease was the same as that observed after treatment with BAM7 together with R-Ras knockdown in M cells. These results indicated that endogenous RasGRP2 behaved similarly to exogenous RasGRP2.

### RasGRP2 inhibits its translocation without affecting the Bax signaling pathway

**Figure 4:**
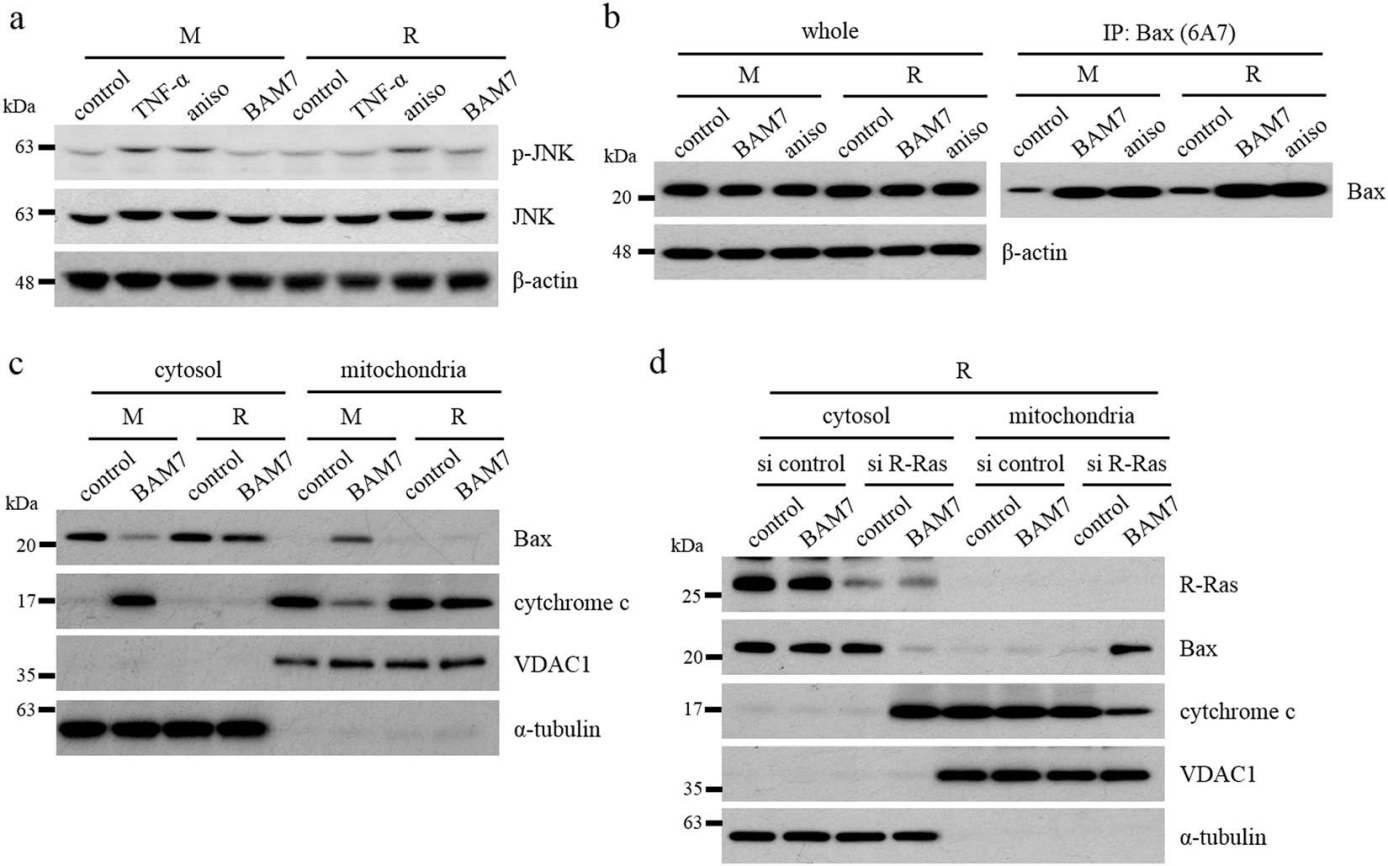
Inhibition of Bax translocation by RasGRP2 via R-Ras-PI3K-Akt signaling pathway. (**a**–**d**) Each effect was detected by western blotting. (**a**) Phosphorylation of JNK was detected using a specific antibody. Cells were incubated with or without TNF-α, anisomycin, or BAM7 for 1 h. (**b**) Activated Bax was isolated by immunoprecipitation (IP) using a Bax (6A7) antibody. Cells were incubated with or without BAM7 or anisomycin for 16 h. (**c**,**d**) Cytosolic and mitochondrial fractions were isolated by cell fractionation kit. (**d**) Cells were pre-treated with siRNAs against R-Ras or negative control siRNA and incubated with or without BAM7 for 16 h. M: mock cells, R: RasGRP2-stable overexpression cells, aniso: anisomycin, IP: immunoprecipitation.

To examine how R cells suppresses apoptosis, we compared the signaling pathways induced by BAM7 and anisomycin in each cell line. As a control experiment, the phosphorylation of c-jun N-terminal kinase (JNK) was increased after treatment with TNF-α in M cells but not in R cells (Fig. 4a). This was because RasGRP2 mediated inhibition of ROS production by NOX inhibition via Rap1 activation. On the other hand, the phosphorylation status of JNK in the both cells were not altered by treatment with BAM7, which directly activates Bax, but were increased by treatment with anisomycin. As a result, no significant difference by treatment with BAM7 or anisomycin was observed between both the cells (Fig. 4a). Furthermore, during the immunoprecipitation experiment using a Bax (clone 6A7) antibody that detects conformationally active Bax, the activation of Bax increased after treatment with BAM7 or anisomycin, and a significant difference was not observed between both the cells (Fig. 4b).

On the other hand, the release of cytochrome c (cyt c) increased, which causes caspase activation, from mitochondria to cytosol after treatment with BAM7 or anisomycin in M cells, while it was inhibited in R cells. Furthermore, the translocation of Bax, from cytosol to mitochondria, was also increased in M cells, while it was suppressed in R cells (Fig. 4c). In addition, mitochondrial Bax and cyt c releases were significantly increased after treatment with BAM7 along with R-Ras knockdown compared to after treatment with BAM7 in R cells (Fig. 4d). A similar result was also obtained after LY294002 treatment (Fig. S5a). In addition, Mcl-1 apoptosis regulator, BCL2 family member (Mcl-1), which is anti-apoptotic member of Bcl-2 family, was degraded after treatment with BAM7 in M cells, while its degradation was inhibited in R cells (Fig. S5b). Mcl-1 was detected only the mitochondrial fraction (data not shown). Furthermore, BAM7-induced Mcl-1 degradation was inhibited by pre-treatment with Q-VD-OPH (Fig. S5b). From these results, it was shown that RasGRP2 suppresses apoptosis by inhibiting Bax translocation from the cytoplasm to mitochondria, via R-Ras-PI3K-Akt signaling pathway.

### RasGRP2 suppresses apoptosis by inhibiting Bax translocation via promotion of translocation of hexokinase-2

First, in order to confirm whether Akt directly phosphorylates Bax, phosphorylation analysis of Bax was performed using Phos-tag SDS-polyacrylamide gel electrophoresis (SDS-PAGE). In the whole cell lysates, using a Bax antibody, non-specific bands were shifted upward; however, the band of immunoprecipitation-purified Bax protein was not changed at all (Fig. S6a). In addition, the phosphorylated Bax could not be also detected by western blot analysis using the anti-p-Bax (Ser184) antibody (data not shown). These results indicated that Bax is not phosphorylated by Akt, and it suggested that Akt does not directly interacts with Bax.

**Figure 5:**
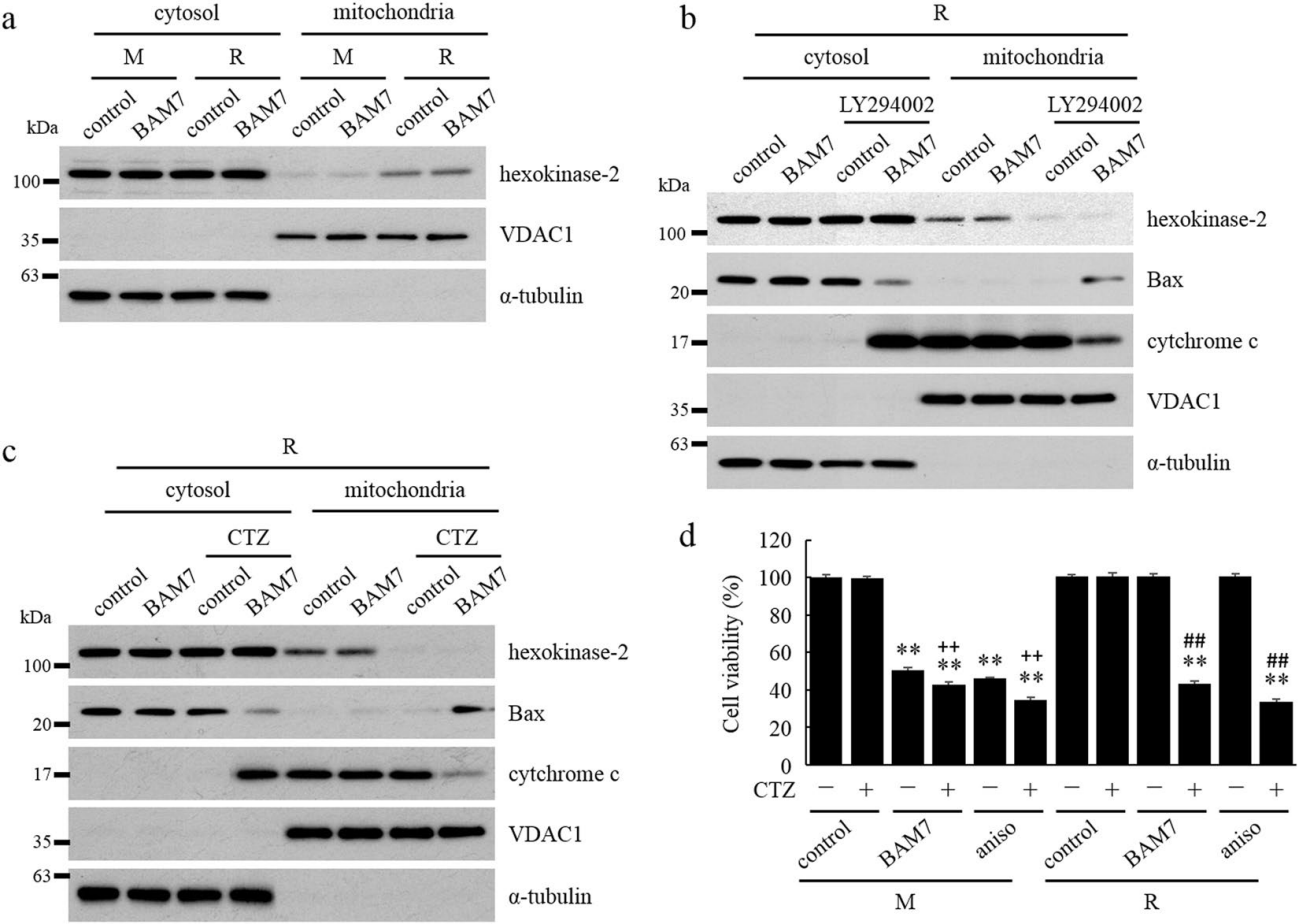
Suppression of apoptosis by promoting mitochondrial hexokinase-2 translocation via RasGRP2-R-Ras-PI3K-Akt signaling pathway. (**a**–**c**) Cells were pre-incubated with or without LY294002 (**b**) or CTZ (**c**) and incubated with or without BAM7 for 16 h. Each effect was detected by western blotting. Cytosol and mitochondrial fractions were isolated by cell fractionation kit. (**d**) Cells were pre-incubated with or without CTZ and incubated with or without BAM7 for 48 h. Cell viability was determined by Cell Counting Kit-8 assay. M: mock cells, R: RasGRP2-stable overexpression cells, CTZ: clotrimazole, aniso: anisomycin. Data are shown as the mean ± SD (n = 3), **P < 0.01 compared with each cell control, ^##^*P < *0.01 compared with each R cell without pre-treatment, and ^++^*P < *0.01 compared with each M cell without pre-treatment.

Next, we analyzed the indirect effects of Bax by Akt. Hexokinase-2 (HK-2), which inhibits Bax translocation in mitochondria, was significantly upregulated in mitochondria without changing its total protein content in R cells compared to M cells (Fig. 5a, S6b). Furthermore, mitochondrial HK-2 was significantly decreased after LY294002 treatment, and mitochondrial Bax and cyt c releases were significantly increased after treatment with BAM7 along with LY294002 pre-treatment compared to those after treatment with BAM7 only in R cells (Fig. 5b). A similar result was also obtained by R-Ras knockdown (Fig. S6c). Under the same conditions, the expression level and localization of hexokinase-1, having the same action as HK-2, did not change (Fig. S6b,d).

Finally, we assessed whether Bax translocation inhibition is dependent on mitochondrial HK-2 using clotrimazole (CTZ), which is a translocation inhibitor of HK-2. Similar to LY294002 treatment, mitochondrial HK-2 was significantly decreased by CTZ treatment, and mitochondrial Bax and cyt c releases were also significantly increased after treatment with BAM7 along with CTZ pre-treatment compared to by treatment with BAM7 alone in R cells (Fig. 5c). In addition, the cell viability of R cells was significantly reduced by treatment with BAM7 along with CTZ pre-treatment to the same extent as M cells (Fig. 5d). These results indicated that phosphorylation of Akt by RasGRP2 suppresses apoptosis by inhibiting Bax translocation via promotion of translocation of HK-2 from cytoplasm to mitochondria.

## Discussion

Apoptotic pathways are roughly divided into mitochondrial pathway, which is the intrinsic pathway, and death receptor pathway, which is the extrinsic pathway. The mitochondrial pathway was induced as a response to cellular stress and results in the activation of the pro-apoptotic BH3-only proteins, such as Bim. Furthermore, BH3-only protein directly or indirectly activates Bax and leads to apoptosis. While, the death receptor pathway is initiated by binding with death ligands, such as TNF-α and cell surface receptors, resulting in apoptosis by activation of caspase-8^28^. BAM7 is a BH3 domain analog of Bim, and is a low molecular weight compound that activates Bax by direct interaction with its trigger site to promote polymerization^29^. In addition, anisomycin is a protein synthesis inhibitor, and is known to activate Bax by phosphorylating JNK and Bim and induce apoptosis^30^. Both compounds were found to cause apoptosis without affecting ROS production in the TERT HUVECs (Fig. 1, S3a,b) and similar results were also obtained in the HUVECs (data not shown). In a previous report, we reported that Rap1 mediated suppression of RasGRP2 by TNF-α-induced apoptosis via ROS production in the TERT HUVECs^27^. Therefore, these compounds were suitable for analyzing the novel mechanism of RasGRP2, and we showed that RasGRP2 completely suppressed the induction of apoptosis by BAM7 and anisomycin (Fig. 1c). Interestingly, this suppression was not ameliorated by the knockdown of Rap1 protein (Fig. 1f). These results suggested that RasGRP2 not only suppresses apoptosis by suppression of ROS production via Rap1, and through another mechanism, can suppress apoptosis without Rap1.

Ohba *et al*. reported that R-Ras, TC21, and Rap2 were slightly activated in 293 T cells transfected with both RasGRP2 and each expression vector^31,32^. We showed that RasGRP2 activates endogenous R-Ras and TC21, but not Rap2A in the TERT HUVECs, and that in M cells and R cells, R-Ras was markedly activated than TC21 (Fig. 2a,b). Furthermore, TC21 did not affect the suppression of apoptosis by RasGRP2 (Fig. S4). These results suggested that R-Ras is a small GTPase that is as important as Rap1 as a target of RasGRP2 in the endothelial cells. R-Ras is classified into the Ras family, but its function is different from H-Ras, N-Ras, and K-Ras, which are the classical Ras^33^, and is known to be highly expressed in the vascular endothelial cells^34^. In the vascular endothelial cells, R-Ras signaling promotes lumenogenesis, supports the lumen structure with the stabilized microtubules^35^, and promotes vessel maturation by endothelial barrier stabilization and pericyte association^36,37^. Komatsu *et al*. demonstrated that the overexpression of R-Ras38V, which is the activated form of R-Ras, in the HUVECs suppressed apoptosis induction by serum starvation, along with suppression of proliferation^34^. Similarly, we showed that R-Ras activated by RasGRP2 suppresses BAM7-induced apoptosis by the experiment of R-Ras knockdown and PI3K inhibitor (Fig. 2f,g). However, no difference in proliferation was found between M cells and R cells (Fig. 1a). This may be due to the difference between the overexpression of R-Ras38V and the activation of endogenous R-Ras by RasGRP2. R-Ras signaling is known to activate Akt via PI3K pathway^35^. We also confirmed phosphorylation of Akt on Thr308 by activation of R-Ras (Fig. 2c). Interestingly, when several monoclonal anti-pSer473 Akt antibodies were used, phosphorylation of Akt on Ser473 remained unchanged (Fig. 2c and data not shown). Partial phosphorylation of Akt on Thr308 but not on Ser473 is also reported to be associated with apoptosis suppression^38^. The present study suggested that phosphorylation of Akt on Thr308 by RasGRP2-activated R-Ras plays an important role in suppressing apoptosis in the HUVECs.

We showed that endogenous RasGRP2 activates endogenous R-Ras and inhibits BAM7-induced apoptosis in TERT HUVECs (Fig. 3). As reported in our previous paper, TERT HUVECs express endogenous RasGRP2 to the same degree as HUVECs. This suggested that endogenous RasGRP2 is involved in apoptosis suppression in endothelial cells^27^. We have also shown that endogenous RasGRP2 expression is regulated, i.e., among the three alternative splicing variants, *rasgrp2* mRNA, containing the distal first exon of the 5′-untranslated region, was expressed in human umbilical artery endothelial cells. Furthermore, a luciferase assay showed that not only the promoter, but also the silencer region, is upstream of the distal first exon, and demonstrated that POU domain, class 2, transcription factor 1 (OCT1/POU2F1) bound to the silencer region using a gel super shift assay^26^. These results suggested that RasGRP2 expression is regulated by a combination of transcriptional promotion and repression. By contrast, changes in its activity induced by post-translational modification have also been reported. Subramanian *et al*. reported that RasGRP2 was strongly phosphorylated on Ser587 and weakly phosphorylated on Ser116/Ser117 by protein kinase A and that phosphorylation of RasGRP2 on Ser587 almost attenuates Rap1 activation^39^. The effect of endogenous RasGRP2 is thought to change significantly with increased its expression and activity. However, signals related to increased endogenous RasGRP2 expression or its GEF activity have not yet been clarified, and further investigation is necessary in the future.

**Figure 6:**
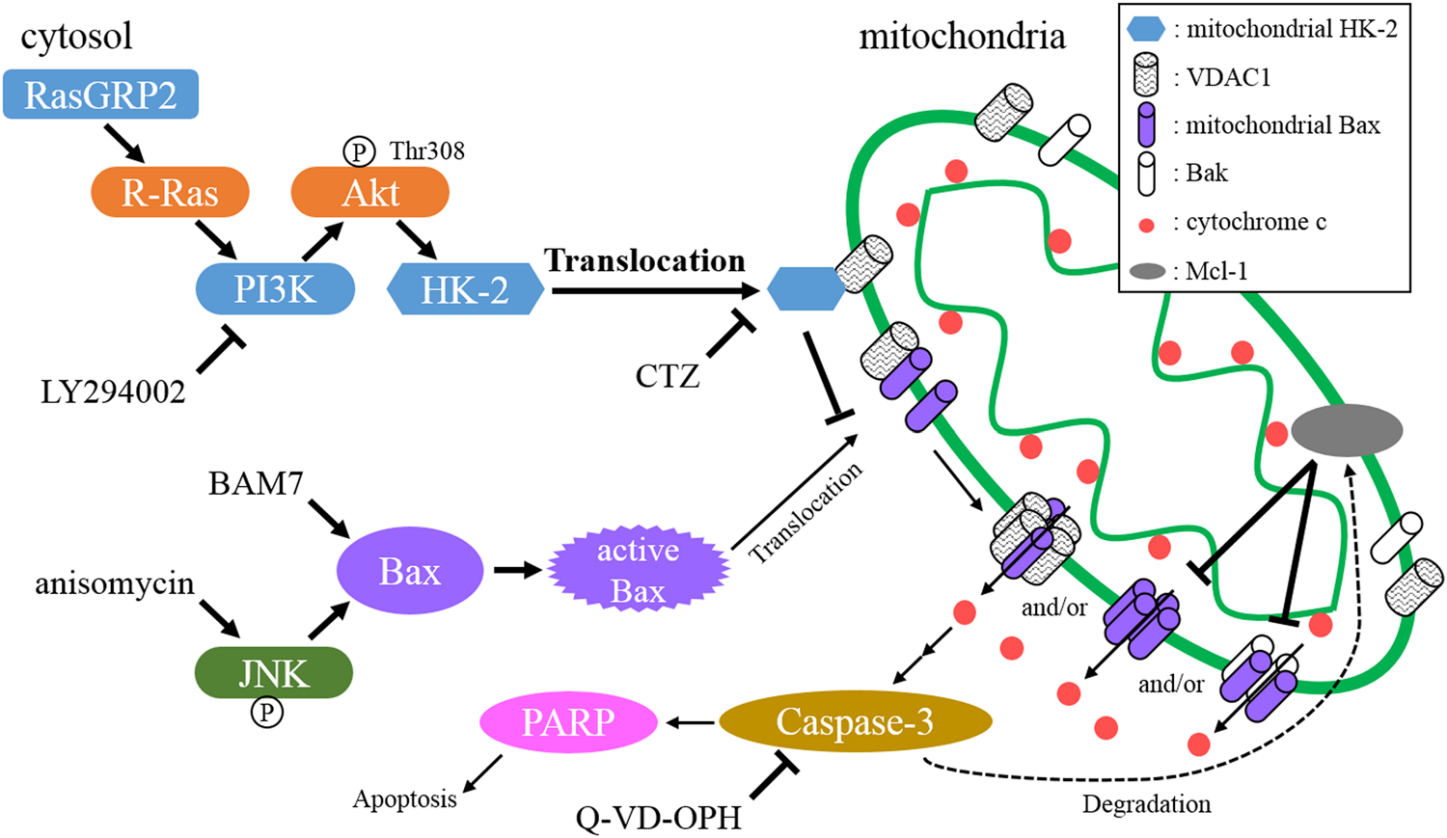
Proposed model for apoptosis suppression via R-Ras pathway by RasGRP2. RasGRP2: ras guanyl nucleotide releasing protein 2, PI3K: phosphoinositide 3-kinase, JNK: c-jun N-terminal kinase, HK-2: hexokinase-2, VDAC: voltage-dependent anion channel, CTZ: clotrimazole, PARP: poly (ADP-ribose) polymerase, Q-VD-OPH: quinoline-Val-Asp-Difluorophenoxymethylketone.

We demonstrated that suppression of BAM7- and anisomycin-induced apoptosis by RasGRP2 was mediated via Bax translocation inhibition (Figs 4 and 5). Activated Bax is translocated from cytosol to mitochondria by binding to voltage-dependent anion channel (VDAC) or binding to its outer membrane. Mitochondrial Bax releases cyt c by the formation of VDAC-Bax oligomer, Bax oligomer, and Bax/Bak pore^40^. The relationship between Akt activation and Bax translocation inhibition from cytosol to mitochondria has been reported in numerous models in various cells but not in vascular endothelial cells^41–47^. With respect to the inhibition of Bax translocation to mitochondria, Gall *et al*. and Pastorino *et al*. demonstrated that mitochondrial HK-2 inhibits Bax translocation without inhibiting Bax activation^48,49^. These reports supported our results because BAM7 used in our study directly activated Bax. HK-2 on Thr473 is phosphorylated by Akt, and this phosphorylation promotes the translocation of HK-2 from the cytosol to mitochondria^41^. HK-2 is also known to bind to VDAC via N-terminal^49^. Furthermore, RasGRP2 also inhibited Mcl-1 degradation induced by BAM7 stimulation (Fig. S5b). However, Mcl-1 degradation caused by BAM7 stimulation occurs after caspase activation and is therefore part of a downstream apoptotic signaling pathway (Fig. S5c). Mcl-1 inhibits the oligomerization of translocated Bax and the activation of Bak^50^; therefore, its degradation by caspase-3 might further promotes apoptosis^51^. Our results suggested that RasGRP2 suppresses the Bax activation-induced apoptosis by promoting HK-2 translocation to mitochondria via R-Ras-PI3K-Akt signaling pathway (Fig. 6). Indeed, the Bax pathway is involved in apoptosis in endothelial cells in conditions of hyperglycemia and methylglyoxal as a trigger of atherosclerosis and in lipopolysaccharide-induced apoptosis caused by inflammation^5,52,53^. Therefore, the inhibition of Bax translocation by RasGRP2 via HK-2 might result in a survival benefit in these conditions.

Taken together with the Rap1-ROS inhibitory pathway that we have already reported^27^, it was suggested that RasGRP2 is involved in complex apoptosis suppression signaling by activating small GTPases, like Rap1 and R-Ras, in the endothelial cells. It may play a very important role in the environment where endothelial cells are exposed to various factors. However, the other effects of RasGRP2 are not yet well understood. In the future, further analysis of RasGRP2 may be useful for development of novel therapeutic strategies for vascular endothelial cell dysfunction.

## Methods

### Reagents

All reagents were commercially available, of high purity and used as supplied. BAM7 was purchased from Adooq BioScience, anisomycin was purchased from Alomone Labs, TNF-α was purchased from PeproTech, NAC was purchased from Wako, and Q-VD-OPH, DPI, LY294002, and CTZ were purchased from Cayman Chemical.

### Cell cultures

TERT HUVECs were grown in Endothelial Cell Growth Medium (PromoCell) under standard cell culture conditions (humidified atmosphere, 5% CO_2_, 37 °C). Stable cell lines were prepared as previously described^27^, stably transfected TERT HUVECs were grown in Endothelial Cell Growth Medium (PromoCell) in the presence of 100 μg/mL hygromycin B (Wako). Cells (2 × 10^5^ cells/mL) were then seeded in various plates or culture dishes and incubated for 6 h (siRNA experiment) or for 24 h before the start of experiments. To investigate the specific effects of RasGRP2, cells were pre-incubated with or without 20 μM Q-VD-OPH, 5 mM NAC, 20 μM DPI, 10 μM LY294002, or 20 μM CTZ for 2 h, and incubated with or without 20 μM BAM7 (for endogenous RasGRP2 analysis, 10 and 20 μM were used), 1 μM anisomycin, or 20 ng/mL TNF-α.

### Knockdown by siRNA

Rap1 siRNA (90 nM; SASI_Hs01_00040403), R-Ras siRNAs (90 nM; EHU022511, siRNA mix), RasGRP2 siRNA (180 nM; SASI_Hs02_00319692), TC21 siRNAs (90 nM; EHU108181, siRNA mix), or negative control siRNA (90 or 180 nM; SIC-001) (SIGMA Aldrich) were transfected into cells, according to the manufacturer’s instructions. Briefly, the cells were seeded, and after 6 h, medium was changed to 10% FCS Endothelial Cell Growth Medium 2 (PromoCell) without heparin. Then, a mixture of MISSION® siRNA Transfection Reagent (SIGMA Aldrich) and siRNA were pre-incubated for 10 min and transfection was performed.

### Cell viability

After the cells were incubated for 24 h to 48 h, 10 µL/well of Cell Counting Kit-8 assay solution (Dojindo Laboratories) was added and incubated for 2 h. Absorbance was then measured at 450 nm and 650 nm using a microplate reader. The net difference in absorbance (A450 – A650) was used as a measure of cell viability.

### Measurement of apoptosis and intracellular ROS

For the measurement of apoptosis, after cells were incubated for 24 h, 1 μM NucView 488 (Biotium Inc.) was added and incubated for 30 min. During the measurement of intracellular ROS, after cells were incubated for 4 h, 5 μM CellROX® Green (Thermo Fisher Scientific) was added and incubated for 1 h. Then, the medium was exchanged and the fluorescence image was acquired. Cells in which apoptosis was induced were measured using ImageJ, and the fluorescence area was shown by average value of three randomly selected fields.

### Measurement of caspase-3/7 activity

After cells were incubated for 24 h, an equal volume of Caspase-Glo 3/7® Reagent (Promega) was added and incubated for 2 h. The luminescence of each sample was then measured using a luminometer.

### Preparation of cell lysate and western blot analysis

Cells were lysed using an IP Lysis Buffer containing Halt Protease & Phosphatase Inhibitor (Thermo Fisher Scientific), centrifuged at 12,000 g for 10 min at 4 °C, and the supernatant was recovered. Protein concentrations were measured using a Bradford assay (Bio-Rad Laboratories). Cell lysates dissolved in LDS sample buffer containing 10% sample reducing agent (Thermo Fisher Scientific), boiled for 10 min at 70 °C, separated by SDS-PAGE, and then, electro-transferred onto polyvinylidene difluoride (PVDF) membranes (Millipore). Membranes were blocked for 1 h at 37 °C using a PVDF blocking reagent for Can Get Signal® (Toyobo). After washing with PBS containing 0.05% Tween 20 (PBS-T), the membranes were incubated with rabbit anti-PARP antibody (GeneTex, GTX100573 at 1:1,500), rabbit anti-RasGRP2 antibody (GeneTex, GTX108616 at 1:5,000), mouse anti-β-actin antibody (Santa Cruz, sc-47778 at 1:12,000), rabbit anti-Rap1A antibody (Santa Cruz, sc-398755 at 1:1,000), mouse anti-p-Akt (Thr308) antibody (Santa Cruz, sc-271966 at 1:6,000), mouse anti-p-Akt (Ser473) antibody (Santa Cruz, sc-81433 at 1:500), mouse anti-Akt antibody (Santa Cruz, sc-81434 at 1:2,000), rabbit p-JNK antibody (Cell Signaling Technology, #4671 at 1:2,000), rabbit JNK antibody (Cell Signaling Technology, #9258 at 1:2,500), mouse anti-R-Ras antibody (Santa Cruz, sc-166221 at 1:100), mouse anti-TC21 antibody (Santa Cruz, sc-166262 at 1:100), rabbit anti-Rap2A antibody (GeneTex, GTX108831 at 1:500), mouse anti-Bax antibody (Santa Cruz, 2D2: sc-20067 at 1:200), rabbit anti-α-tubulin 4a antibody (GeneTex, GTX112141 at 1:1,000), mouse anti-VDAC-1 antibody (Santa Cruz, sc-390996 at 1:1,000), mouse anti-cytochrome c antibody (Santa Cruz, sc-13156 at 1:500), rabbit anti-MCL1 antibody (GeneTex, GTX102026 at 1:500), rabbit anti-Hexokinase 2 antibody (GeneTex, GTX111525 at 1:5,000) or rabbit anti-Hexokinase 1 antibody (GeneTex, GTX105248 at 1:3,000) in Can Get Signal® Solution 1 (Toyobo) for 1 h. Subsequently, the membranes were washed thrice with PBS-T and incubated with anti-rabbit IgG antibody (GeneTex, GTX77057 at 1:5,000) or anti-mouse IgG antibody (DakoCytomation, P0260 at 1:5,000) in Can Get Signal® Solution 2 (Toyobo) for 1 h. After five additional washes with PBS-T, immunoreactive proteins were detected using Western BloT Quant HRP or Ultra Sensitive HRP Substrate (Takara) and Amersham hyperfilm ECL (GE Healthcare).

### Small GTPase activity assay

The activity of Rap1A, Rap2A, R-Ras, and TC21 were examined using RalGDS-RBD agarose beads or Raf1-RBD agarose beads (Cell Biolabs). This assay is a pull-down method based on the specific binding of agarose beads to the active, GTP-bound form of small GTPases. Briefly, cell lysates (150 μg) and each RBD agarose beads (40 μL) were incubated for 1.5 h at 4 °C with gentle rotation. The beads were then washed thrice with excess lysis buffer, and bound proteins were eluted in LDS sample buffer containing 10% sample reducing agent, boiled for 10 min at 70 °C.

### Immunoprecipitation

After incubating with 15 μL of Protein G Mag SepharoseTM (GE Healthcare) with 1 μg of rabbit anti-Bax antibody (GeneTex, GTX109683) or mouse anti-Bax antibody (Santa Cruz, 6A7: SC-23959) for 1 h at 4 °C, cell lysates (100 or 150 μg) were captured using complex beads for 1 h at 4 °C. The tubes were then placed on a magnet for 2 min to allow collection of the immunoprecipitates, which were subsequently washed three times with PBS (-). Immunoprecipitates were dissolved in LDS sample buffer containing 10% sample reducing agent, boiled for 10 min at 70 °C.

### Cytosol and mitochondria fraction extraction

The cytosol and mitochondrial fractions were extracted via buffer extraction with a cell fractionation kit (Abcam, ab109719), according to the manufacturer’s protocol.

### Phos-tag SDS-PAGE

Phosphorylated proteins in the LDS sample buffer containing 10% sample reducing agent were separated by Phos-tag SDS-PAGE (5% polyacrylamide gels including 20 μM Phos-tag acrylamide (Wako) and 0.1 mM MnCl_2_), and Mn^2+^ in the gel was removed with transfer buffer containing EDTA. Then, the bands were electro-transferred onto PVDF membranes and immunoreaction assay of the PVDF membrane was conducted as described previously.

### Statistical analysis

All experiments were performed in duplicates and repeated at least two or three times; each experiment yielded essentially identical results. Data are expressed as the mean ± standard deviation (SD). The significance of differences between group means was determined using a one-way analysis of variance, t test. P < 0.05 was defined as statistically significant.

## Supplementary information

Supplementary Information

## Data Availability

The datasets generated during and/or analyzed during the current study are available from the corresponding author on reasonable request.
