# Supplementary material for: The inhibition of Bax activation-induced apoptosis by RasGRP2 via R-Ras-PI3K-Akt signaling pathway in the endothelial cells
Source: Sci Rep. 2019 Nov 13;9:16717. doi: 10.1038/s41598-019-53419-4 (PMC6854084; doi:10.1038/s41598-019-53419-4)
Supplement: Supplementary file 1 — Supplementary Information [file 41598_2019_53419_MOESM1_ESM.pdf]

Supplementary Information

The inhibition of Bax activation-induced apoptosis by RasGRP2 via R-Ras-PI3K-Akt signaling pathway in the endothelial cells

Jun-ichi Takino[1*], Takuma Sato[1], Kentaro Nagamine[1,2], and Takamitsu Hori[1]

[1] Laboratory of Biochemistry, Hiroshima International University, Hiroshima, Japan

[2] Department of Clinical Nutrition, Hiroshima International University, Hiroshima, Japan

*To whom correspondence should be addressed: Jun-ichi Takino: j-takino@hirokoku-u.ac.jp

Included in Supplementary Information:

Supplemental Figures 1-7 and legends

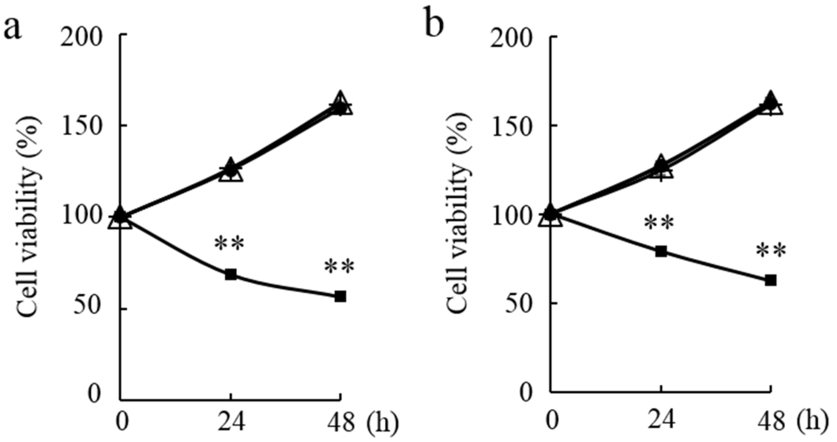

Figure S1. Suppression of apoptosis by RasGRP2 in other clones. Cell viability was determined by Cell Counting Kit-8 assay. Cells were incubated with or without BAM7 (**a**) or anisomycin (**b**) for 24 h to 48 h. Cell viability at 0 h was taken as 100%, circle: M control, square: BAM7 or anisomycin treated M, triangle: R control, cross: BAM7 or anisomycin treated R. M: mock cells, R: RasGRP2-stable overexpression cells. Data are shown as the mean ± SD (n = 3), $^{**}P < 0.01$ compared with each cell control at the same time point.

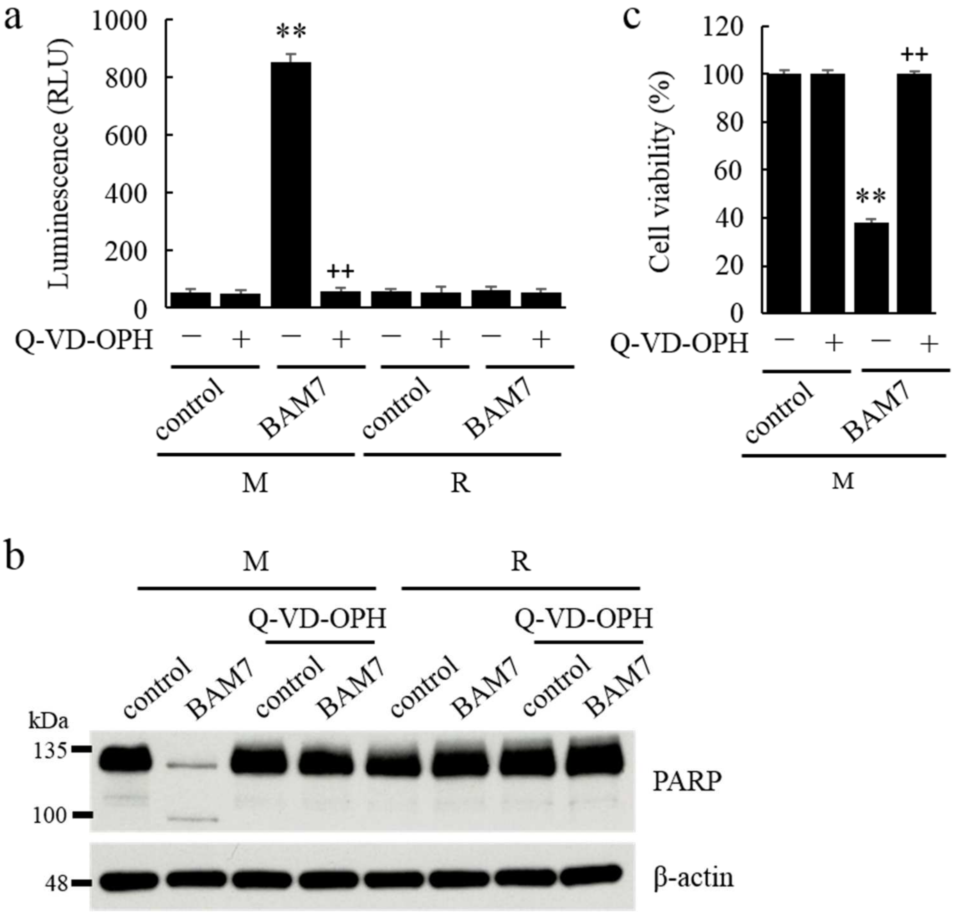

Figure S2. Induction of apoptosis by BAM7 treatment. (**a,b**) Cells were pre-incubated with or without Q-VD-OPH and then incubated with or without BAM7 for 24 h. (**a**) Caspase-3/7 activity was determined using the Caspase-Glo 3/7 Reagent. (**b**) Cleaved PARP was detected using western blotting with a specific antibody. (**c**) Cells were pre-incubated with or without Q-VD-OPH and then incubated with or without BAM7 for 48 h. Cell viability was determined using the Cell Counting Kit-8 assay. M: mock cells, R: RasGRP2-stable overexpression cells. Data are shown as the mean ± SD (n = 3), $^{**}P < 0.01$ compared with each cell control, and $^{++}P < 0.01$ compared with each M cell without pre-treatment.

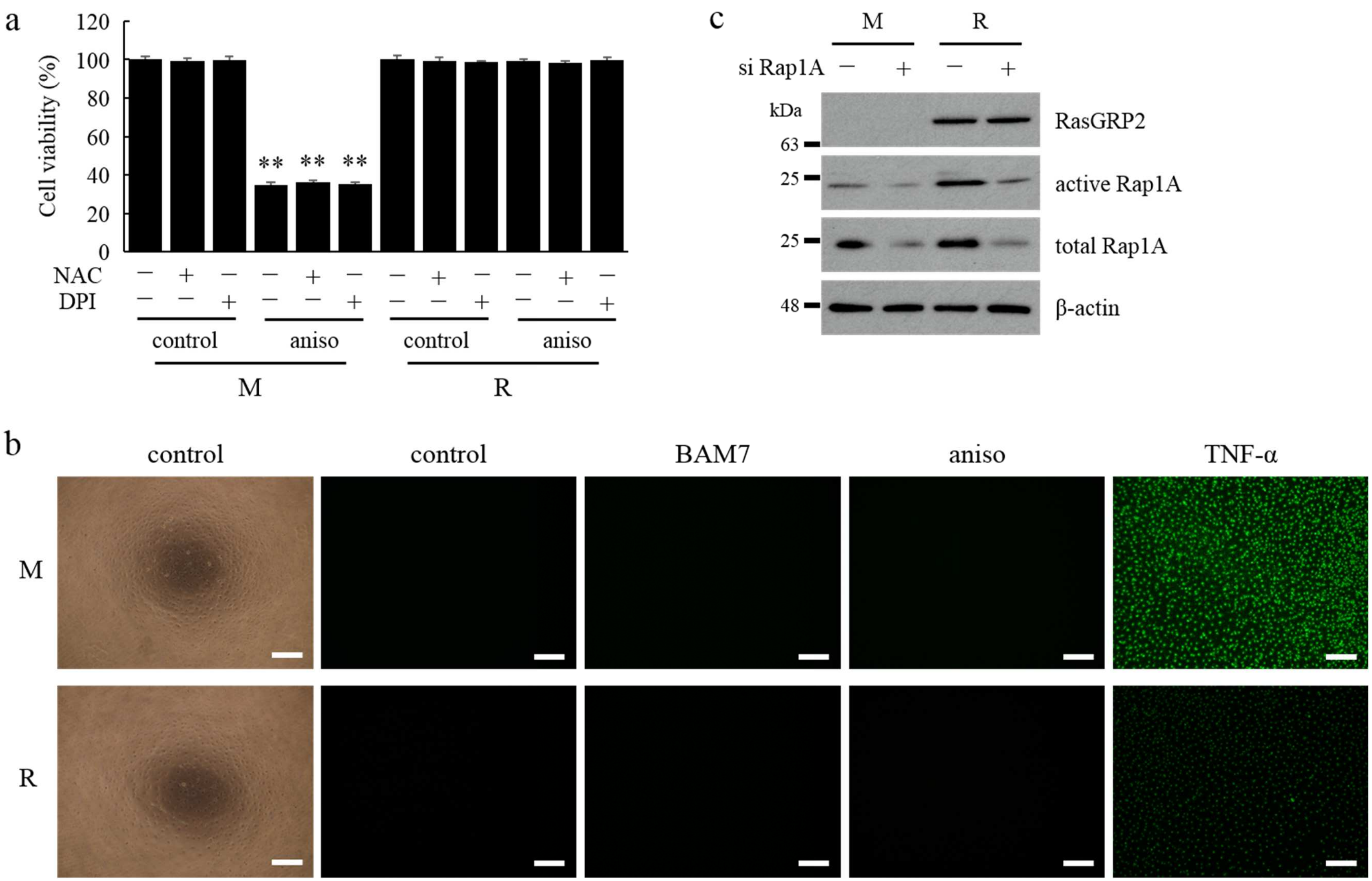
a
Cell viability (%)
120
100
80
60
40
20
0
** ** **
NAC
DPI
control
aniso
control
aniso
M
R
c
M
R
si Rap1A
kDa
63
25
25
48
RasGRP2
active Rap1A
total Rap1A
β-actin
b
control
control
BAM7
aniso
TNF-α
M
R

Figure S3. Induction of apoptosis without ROS production and knockdown of Rap1A protein. (**a**) Cell viability was determined by Cell Counting Kit-8 assay. Cells were pre-incubated with or without NAC or DPI and incubated with or without anisomycin for 48 h. (**b**) Cells were incubated with or without BAM7, anisomycin, or TNF-α for 4 h. Intracellular ROS was determined by CellROX Green. Left panels: bright field image, others: fluorescent image, scale bar = 250 μm. (**c**) Cells were treated with siRNA against Rap1A or negative control siRNA. Activated Rap1A was isolated by pull-down assay using RalGDS-RBD agarose beads, and was detected by western blot assay. M: mock cells, R: RasGRP2-stable overexpression cells, aniso: anisomycin. Data are shown as the mean ± SD (n = 3), $^{**}P < 0.01$ compared with each cell control.

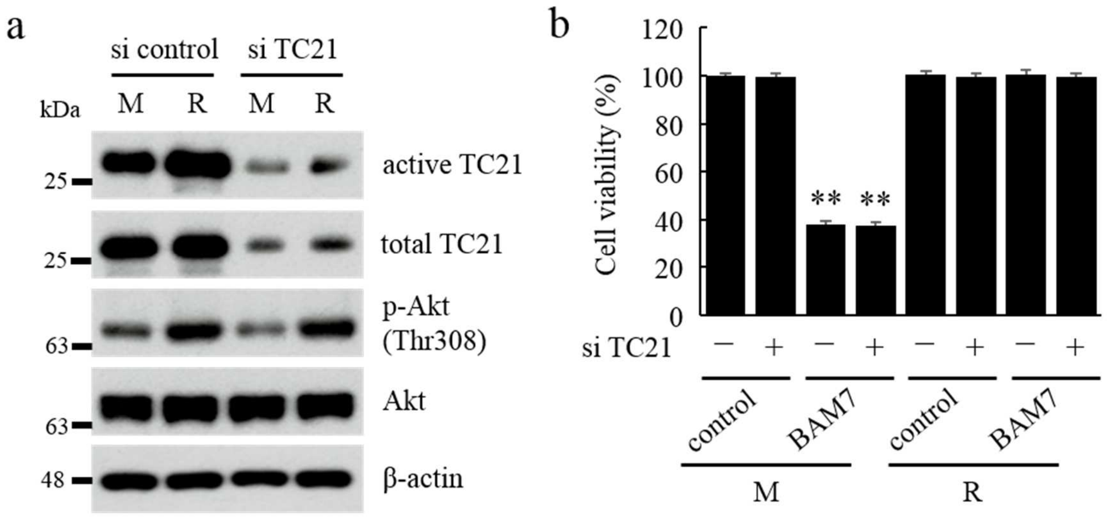

Figure S4. Involvement of TC21 activity in the suppression of apoptosis by RasGRP2. (**a**) Cells were treated with siRNAs against TC21 or a negative control siRNA. Activated TC21 was isolated using a pull-down assay with Raf1-RBD agarose beads. TC21 and phosphorylation of Akt were detected using western blotting with specific antibodies. (**b**) Cell viability was determined using a Cell Counting Kit-8 assay. Cells were pre-treated with siRNAs against TC21 or a negative control siRNA and the incubated with or without BAM7 for 48 h. M: mock cells, R: RasGRP2-stable overexpression cells. Data are shown as the mean ± SD (n = 3), $**P < 0.01$ compared with each cell control.

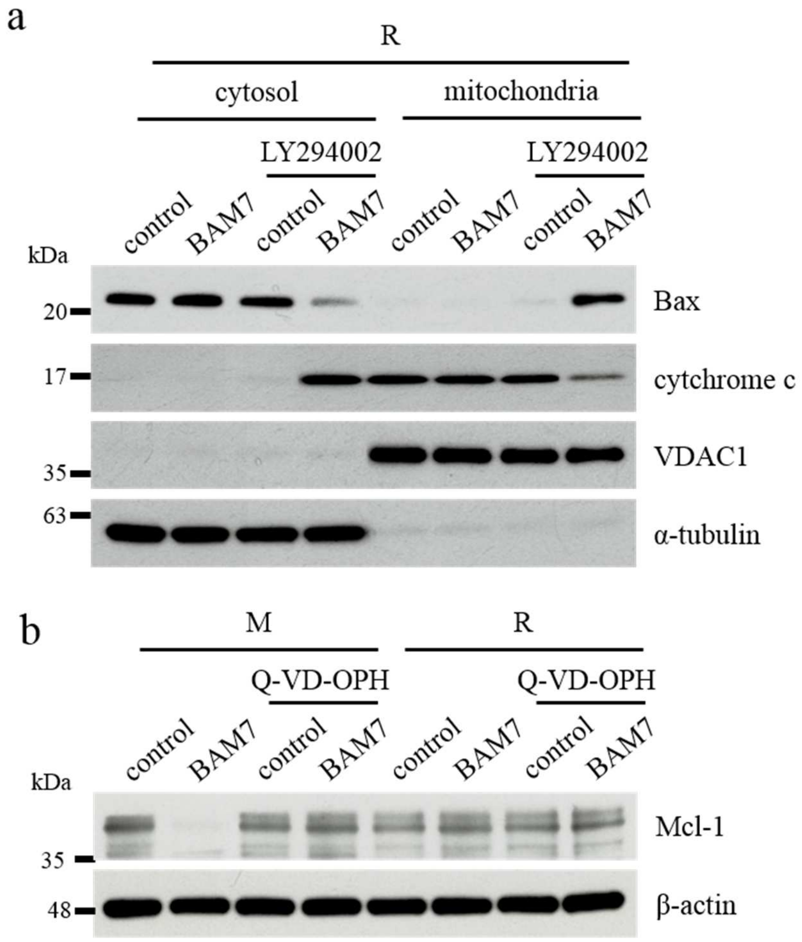

Figure S5. Inhibition of Bax translocation by RasGRP2 via the Akt signaling pathway and detection of Mcl-1 degradation. Each effect was detected using western blotting. (**a**) Cells were pre-incubated with or without LY294002 and then incubated with or without BAM7 for 16 h. Cytosolic and mitochondrial fractions were isolated using a cell fractionation kit. (**b**) Cells were pre-incubated with or without Q-VD-OPH and then incubated with or without BAM7 for 24 h. M: mock cells, R: RasGRP2-stable overexpression cells.

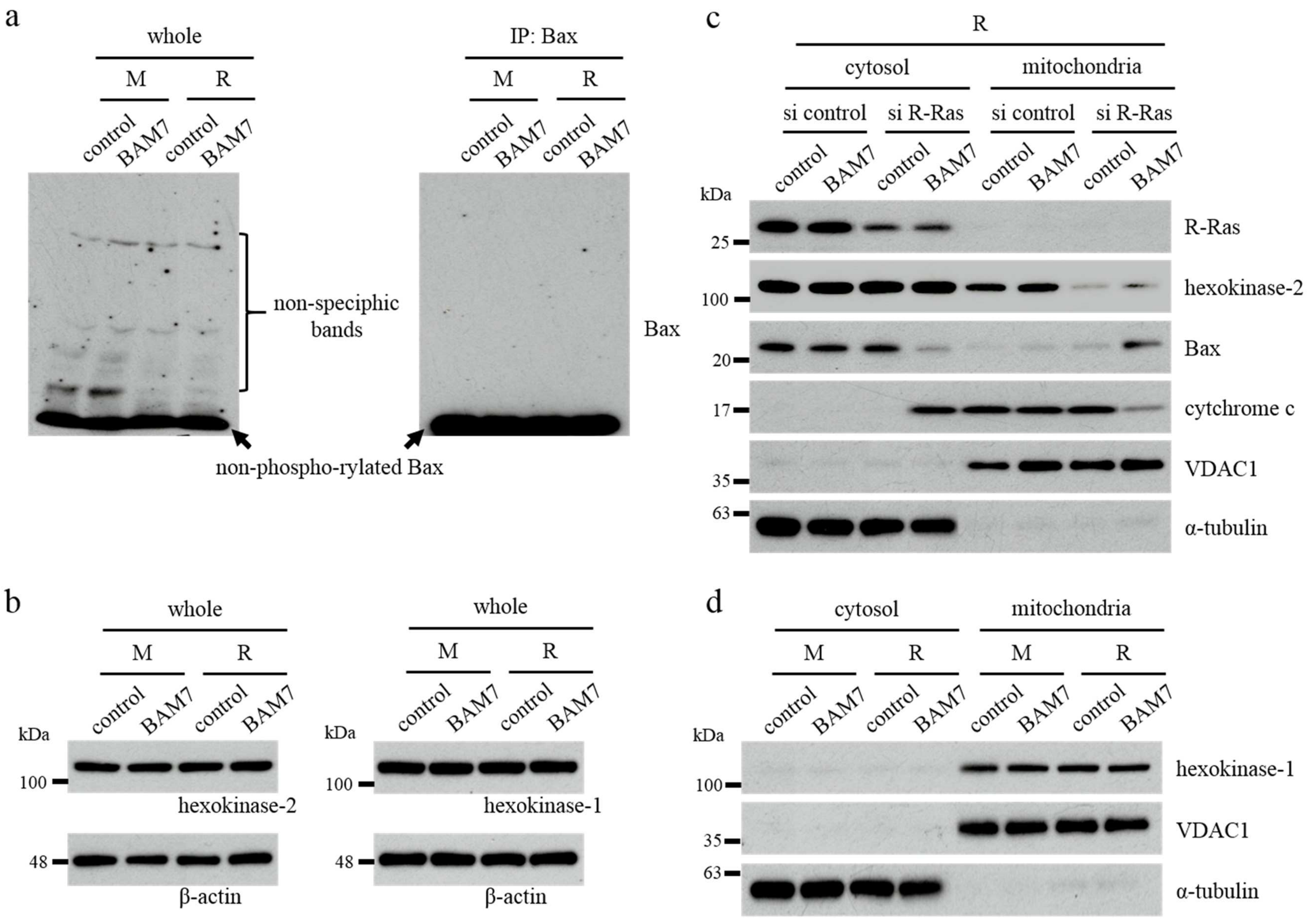

a
whole
M
R
control
BAM7
IP: Bax
non-speciphic bands
non-phospho-rylated Bax
Bax
b
kDa
100
48
hexokinase-2
β-actin
hexokinase-1
c
R
cytosol
mitochondria
si control
si R-Ras
25
20
17
35
63
R-Ras
Bax
cytchrome c
VDAC1
α-tubulin
d

Figure S6. Effects of Bax phosphorylation and hexokinase-1/2 translocation by RasGRP2. (**a,b**) Cells were incubated with or without BAM7 for 16 h. Each effect was detected using western blotting. (**a**) Separation of phosphorylated Bax using Phos-tag SDS PAGE. Bax was isolated using IP with a Bax antibody (GeneTex). (**b**) Total hexokinase-2 and hexokinase-1 proteins were detected using western blotting in a whole cell lysate. (**c,d**) Cytosolic and mitochondrial fractions were isolated using a cell fractionation kit. (**c**) Cells were pre-treated with siRNAs against R-Ras or a negative control siRNA and incubated with or without BAM7 for 16 h. (**d**) Cells were incubated with or without BAM7 for 16 h. M: mock cells, R: RasGRP2-stable overexpression cells, IP: immunoprecipitation.

Figure S7 Unprocessed blot images

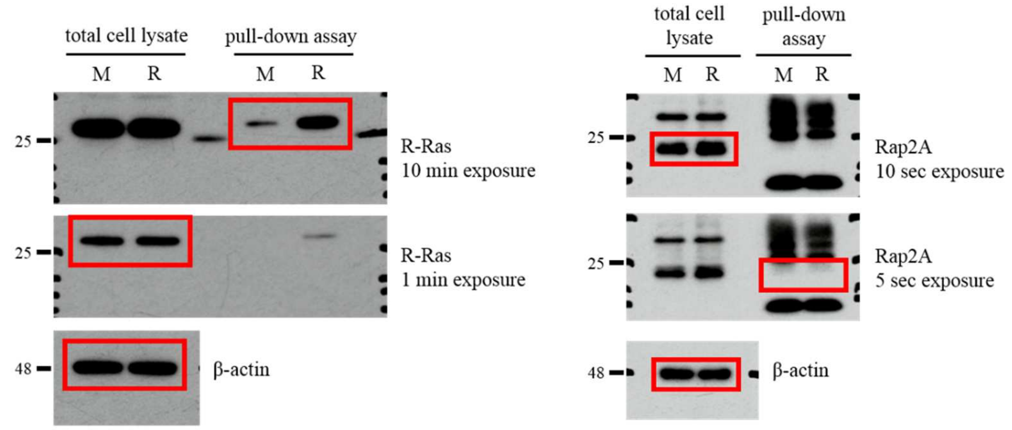

Fig. 2a

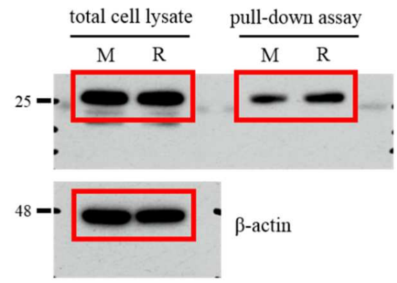

Fig. 2b

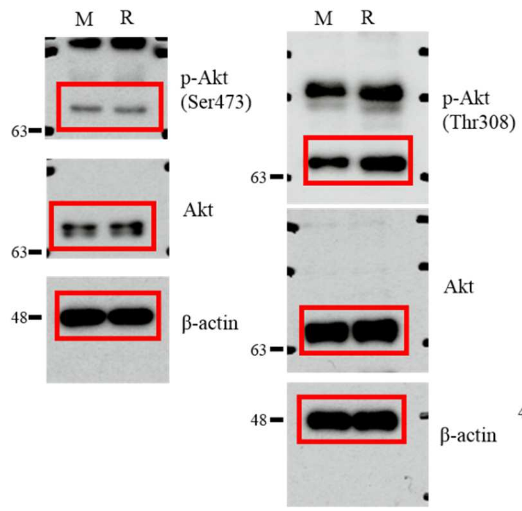

Fig. 2c

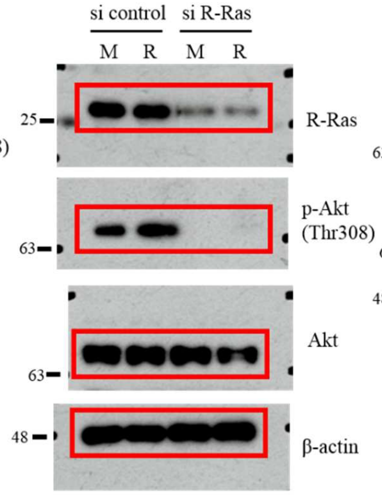

Fig. 2d

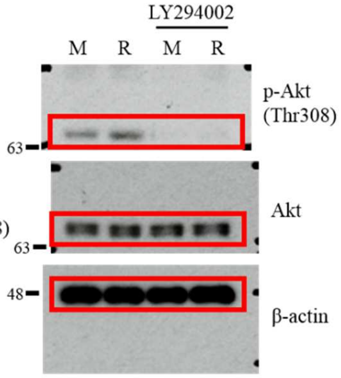

Fig. 2e

Figure S7 Unprocessed blot images (Continued)

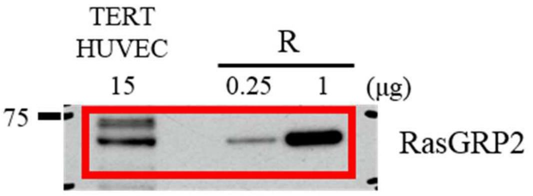

Fig. 3a

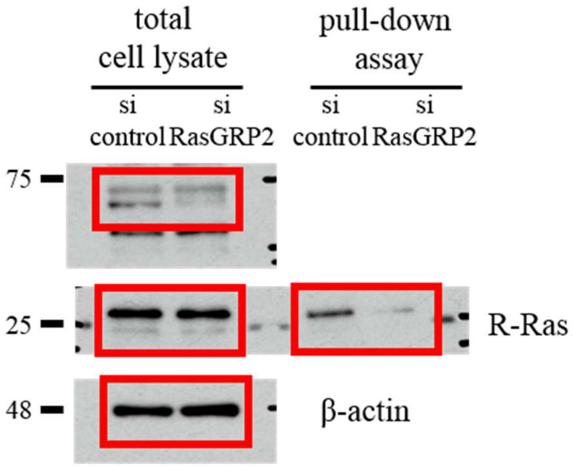

Fig. 3b

Figure S7 Unprocessed blot images (Continued)

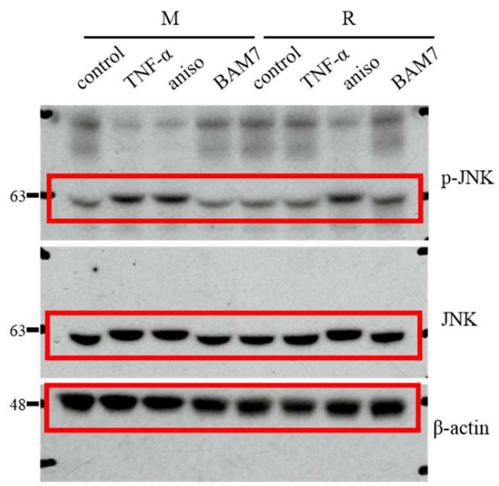

Fig. 4a

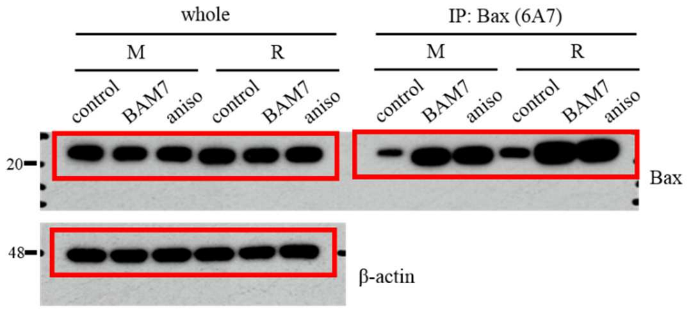

Fig. 4b

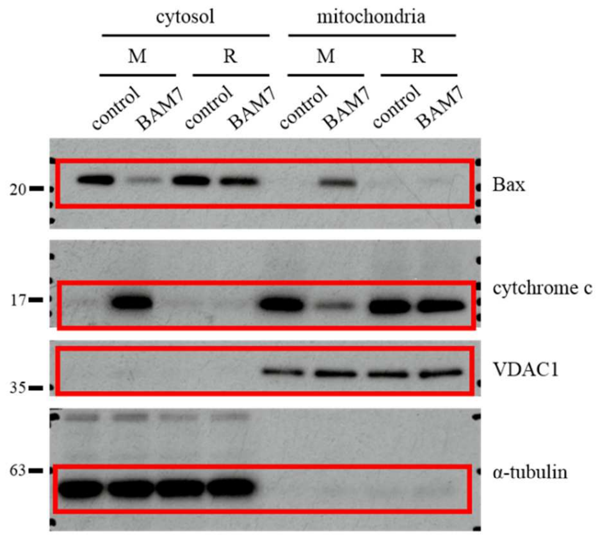

Fig. 4c

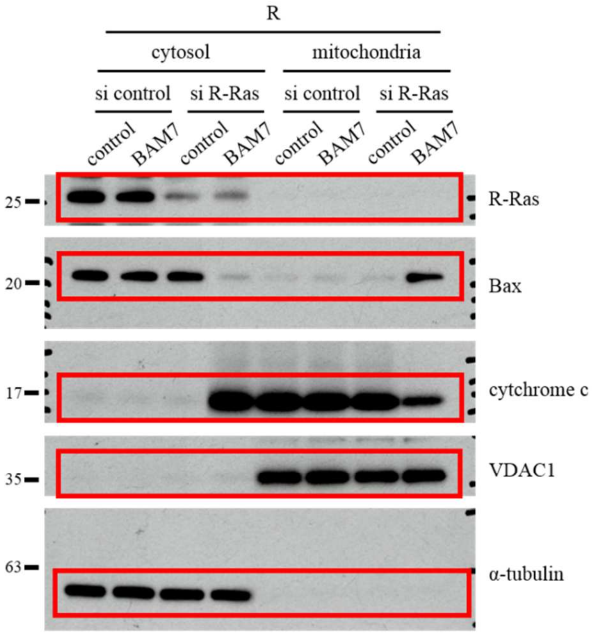

Fig. 4d

Figure S7 Unprocessed blot images (Continued)

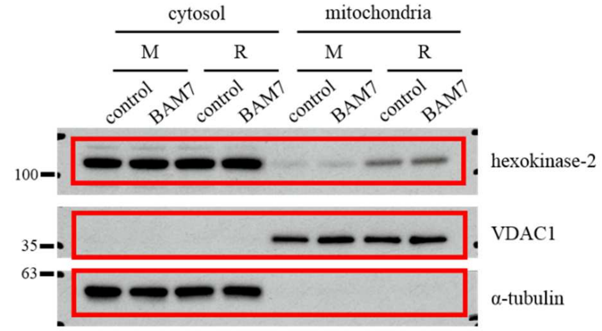

Fig. 5a

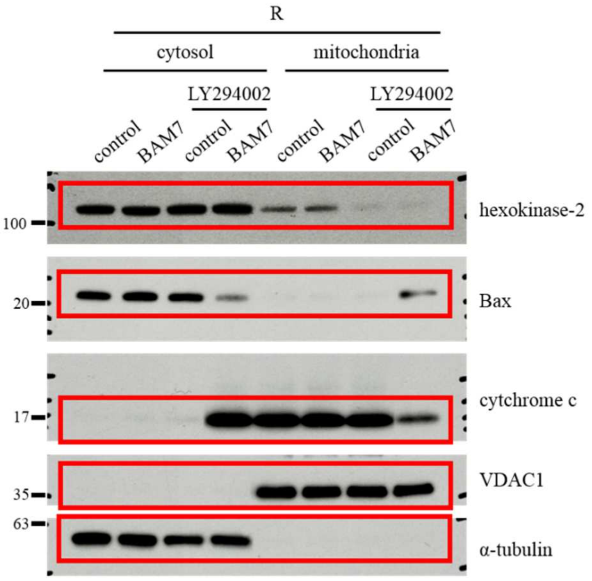

Fig. 5b

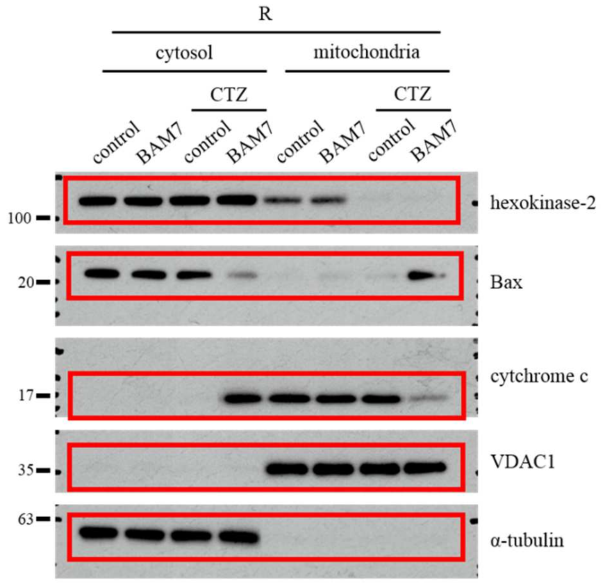

Fig. 5c

Figure S7 Unprocessed blot images (Continued)

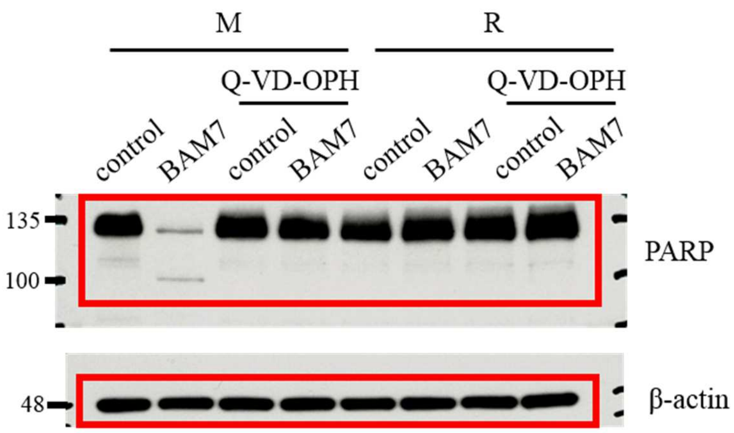

Fig. S2b

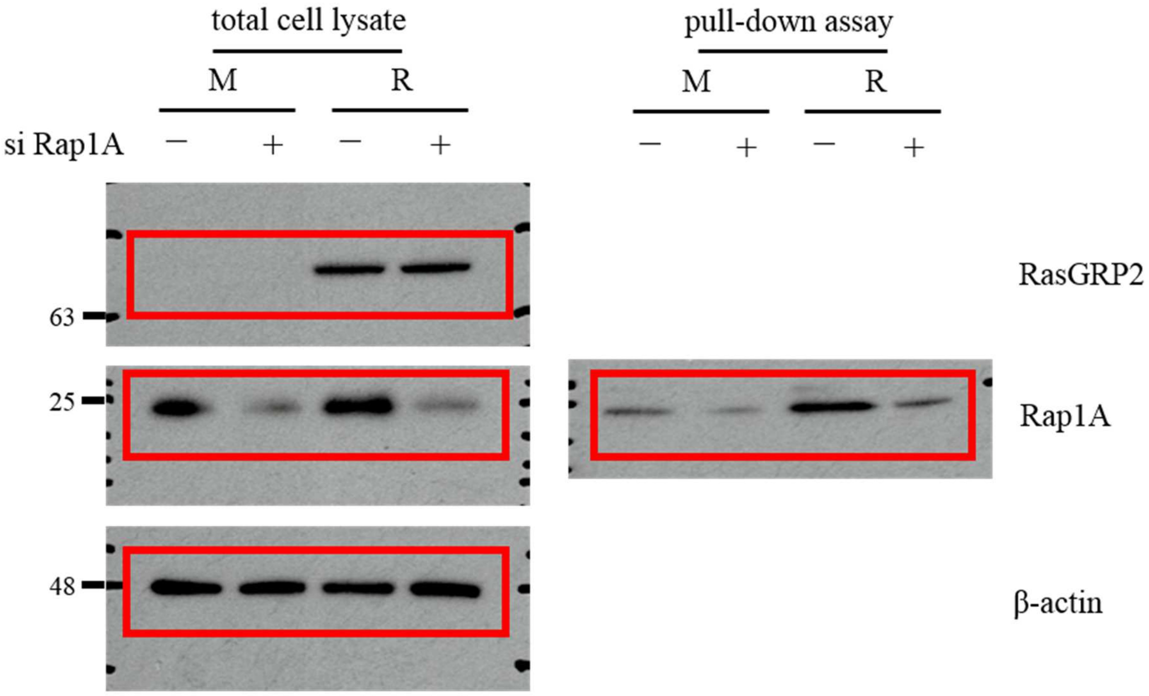

Fig. S3c

Figure S7 Unprocessed blot images (Continued)

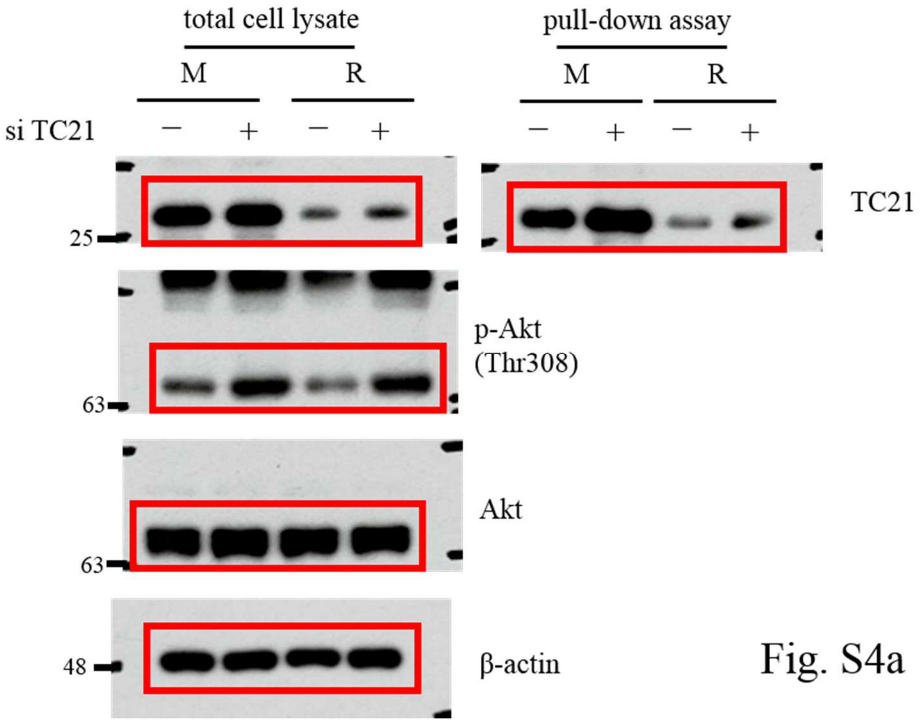

Fig. S4a

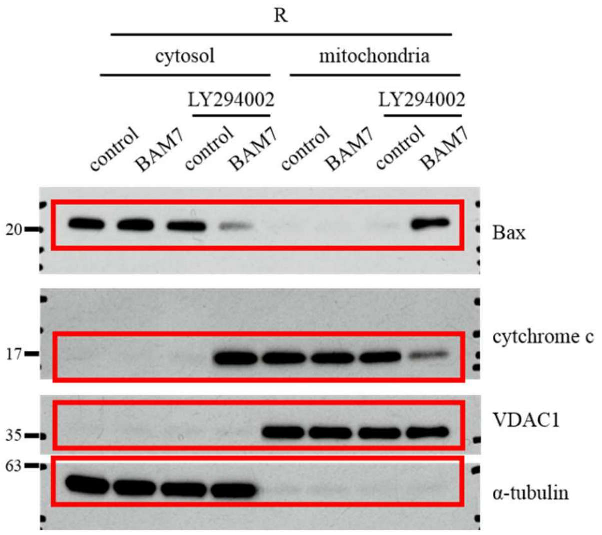

Fig. S5a

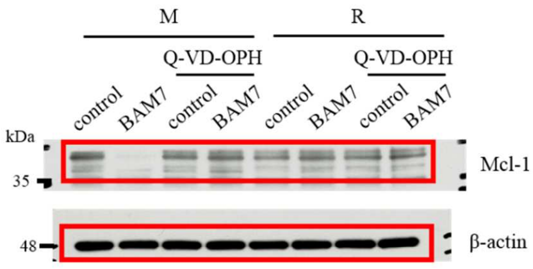

Fig. S5b

Figure S7 Unprocessed blot images (Continued)

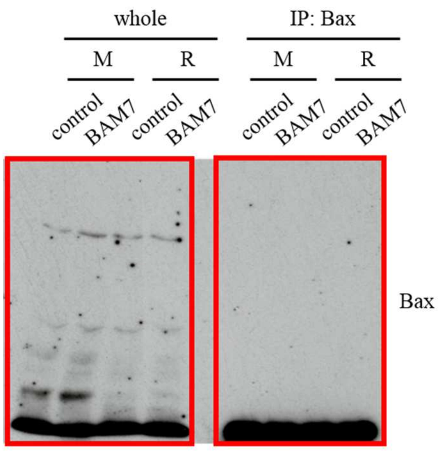

Fig. S6a

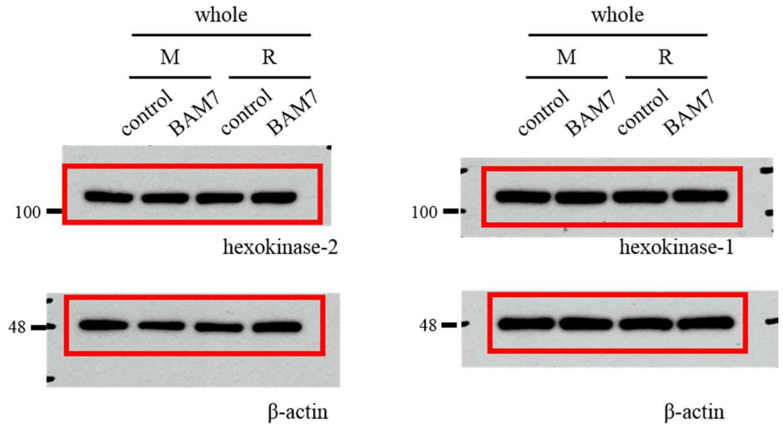

Fig. S6b

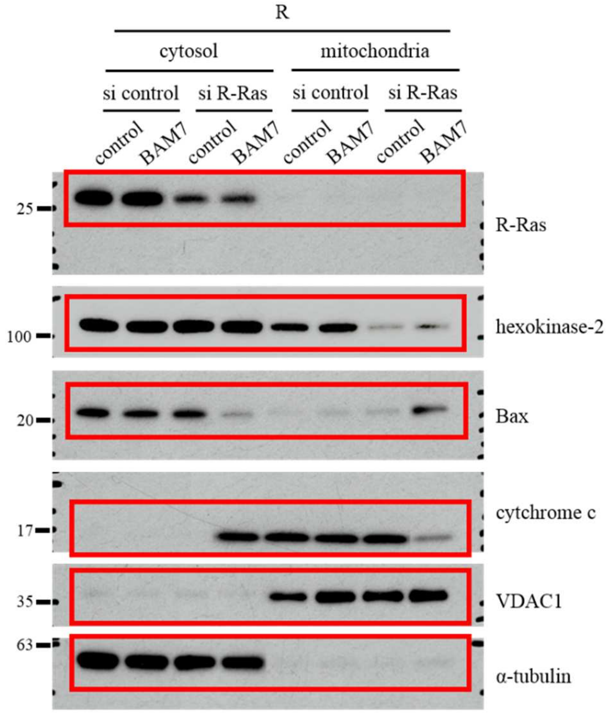

Fig. S6c

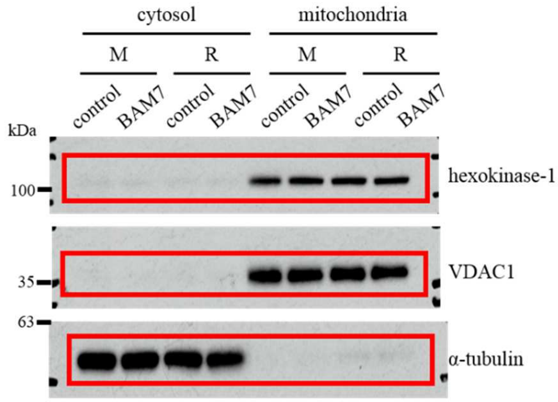

Fig. S6d
